# Schiff Base Ligand 3-(-(2-Hydroxyphenylimino) Methyl)-4H-Chromen-4-One as Colorimetric Sensor for Detection of Cu^2+^, Fe^3+^, and V^5+^ in Aqueous Solutions

**DOI:** 10.1155/2022/4899145

**Published:** 2022-12-30

**Authors:** Ali Q. Alorabi, Sami A. Zabin, Mohammad Mahboob Alam, Mohamed Abdelbaset

**Affiliations:** ^1^Chemistry Department, Faculty of Science, Albaha University, P.O. Box 1988, Albaha 65799, Saudi Arabia; ^2^Chemistry Department, Faculty of Science, Al Azhar University, Assiut Branch, Assiut 71524, Egypt

## Abstract

The ligand 3-(-(2-hydroxyphenylimino) methyl)-4H-chromen-4-one (SL) has been synthesized and examined as a chemosensor for some metal ions in aqueous solutions based on colorimetric analysis. Color changes were monitored using UV–visible spectroscopy. Binding stoichiometry and limit of detection (LOD) were estimated using titration experimentation based on UV–visible absorbance and Job's plot. The synthesized ligand was tested for selectivity in the presence of several cations and was examined for possible utility as a chemosensor in real water samples. The results indicated sensing ability and selectivity for Cu^2+^, Fe^3+^, and V^5+^. Stable complexes were formed between SL and Cu^2+^, Fe^3+^, and V^5+^, and the ligand-to-metal binding stoichiometry was found 2 : 1 in the SL-Cu^2+^ and SL-Fe^3+^ complexes, and 1 : 1 in the SL-V^5+^ complex. The results of LOD and bending constant were (7.03 *μ*M, 1.37 × 10^4^ M^−1^), (5.16 *μ*M, 2.01 × 10^4^ M^−1^), and (5.94 *μ*M, 1.82 × 10^4^ M^−1^) for Cu^2+^, Fe^3+^, and V^5+^, respectively.

## 1. Introduction

It is well established that some metal ions have important beneficial roles and functions in biological and chemical processes, and on the other hand, overexposure to some metal ions causes toxicity. Transition metals are abundant in nature, particularly in soils and aquatic systems and releases of these elements, to the environment are associated with industrial processes. However, their extreme presence in water resources causes serious contamination and can be considered hazardous pollutants at some levels, and creates a threat to human beings and other biota because of their bioaccumulation in food chains and toxicity effects [1, 2]. Therefore, it is very necessary to determine and detect their presence in a water medium to ensure water quality and avoid their adverse effects.

Among the most commonly occurring transition metal ions that may be present in water systems and could potentially cause serious health problems are Cu^2+^, Fe^3+^, and V^5+^. Information of the high levels of exposure to copper from diet and drinking water results in gastrointestinal and hepatic systems illness as it is absorbed rapidly by the stomach and intestine [3]. Iron toxicity is hazardous to children, and subchronic and chronic exposure to iron results in excessive iron absorption and causes hemochromatosis, and studies strongly suggest iron is noxious to tissues [4]. Vanadium is also a toxic metal and a potent environmental pollutant. The primary targets of exposure to vanadium are the gastrointestinal and hematological systems [5].

The detection and recognition of heavy metal ions in different sources is an active field of research and has been a focus of researchers in recent years. Enormous attempts have been devoted for obtaining and developing practical, easy, selective, sensitive, and rapid sensors for the detection of metal ions [1, 2].

Colorimetric sensors that rely on color changes on bounding with the analytes are believed to be one of the most efficient and suitable techniques for detecting inorganic ions and other chemical species [6, 7]. Colorimetric chemosensors-based techniques are one of the interesting tools that can be used for this purpose because of their advantages, such as their ability to produce a desirable on-site“naked-eye” response, a reasonable cost, simplicity, selectivity, a low detection limit, immediate response, and high sensitivity [6–8]. Chemosensor molecules that can be used for sensing the presence of metal ions must have the ability to bind to metal ions and produce detectable physicochemical changes easily and promptly. Recently, there has been a large body of research articles and reviews published reporting the use and development of many molecules that can be used as colorimetric chemosensors for detecting metal ions in different sources [8–10]. One of these interesting compounds is Schiff base ligands, which can coordinate easily with metal ions, forming specific color changes that indicate the presence of specific metal ions [8, 11]. In general, the Schiff base ligands on binding with metal ions produce colored complex compounds that can be recognized easily by the naked eye. Based on this character, these coordination entities can be developed to be used as colorimetric sensors for detecting the presence of metal ions in different sources [1]. The Schiff base ligand 1-(2-thiophenylimino)-4-(N-dimethyl) benzene was investigated by our research group for the detection of multiple metal ions in solutions and showed remarkable selectivity and sensitivity response toward four metal cations: Fe^2+^, Fe^3+^, Cr^3+^, and Hg^2+^ [1]. The Schiff base 2-(3-(2-hydroxyphenylamino)-1,3-diphenylallylidene) amino) phenol was reported by Mergu and Gupta as a highly selective and sensitive chemosensor towards Cu^2+^ ions over other metal ions in a wide pH range [12]. A simple and reversible pyrrole-based Schiff base, (E)-N′-((1H-pyrrol-2-yl) methylene), isonicotinohydrazide chemosensor for the detection of Cu^+2^ ions was reported by Sidana et al., which showed high selectivity and sensitivity by the colorimetric and spectroscopic method in an aqueous organic solvent over other possible interfering ions in a wide pH range. Moreover, the Cu^+2^ detection limit was lower than the WHO-recommended guidelines for drinking water, and practical application for real sample analysis was observed via naked eye color change [13].

Two colorimetric pyrrole-based Schiff base chemosensors were investigated in an aqueous analyte solution by Udhayakumari and Velmathi, which showed highly sensitive probes towards multimetal ions (Fe^+3^, Cu^+2^, Hg^+2^, and Cr^+3^) in water solutions by a distinct color change and exhibited a low detection limit of micromolar levels [14].

Schiff bases derived from 3-formyl chromone have been studied significantly and have attracted the attention of chemists and researchers as they can easily bind with metal ions, forming metal complexes that have many applications [15, 16]. Chromone-based azomethine colorimetric chemosensors were reported by Rezaeian et al. and successfully used to detect and recognize Cu^2+^, Zn^2+^, and CN^−^ ions [17].

In continuation to our research work, herein this investigation we present the design and preparation of the multidentate Schiff base ligand named 3-(-(2-hydroxyphenylimino) methyl)-4H-chromen-4-one by condensing 3-formyl chromone with 2-aminophenol that have the ability to bind to metal ions forming colored complexes and testing it as a colorimetric chemosensor for some metal ions in aqueous solutions.

## 2. Materials and Methods

### 2.1. Materials and Characterization

All chemicals used in synthesis and experimentation were of analytical grade, purchased from Sigma-Aldrich and BDH chemical companies, and were used without purification. The absolute ethanol solvent used in experiments was of HPLC/spectrophotometric grade (97.8%). The precursor compound, 3-formyl chromone, and the Schiff base ligand were synthesized in our laboratory. The synthesized Schiff base ligand was characterized using mass spectroscopy, FT-IR, and ^1^H NMR. The mass spectrum was recorded on a ThermoFisher Scientific-LCQ fleet ion trap mass spectrometer with high resolution using the electrospray ionization (ESI) method. Infrared measurements were made in the 4000–400 cm^−1^ region on a ThermoFisher Scientific Nicolet iS50 FT-IR spectrophotometer using the attenuated total reflection (ATR) method for solid powder. The ^1^ H NMR spectrum was recorded on a Bruker-400 MHz and TMS as an internal standard working in DMSO d^6^, respectively. UV–vis absorption spectra were recorded with a ThermoFisher Scientific Evolution 300 UV–visible double beam spectrophotometer in the range of 200–800 nm.

### 2.2. Preparation of the Precursor Compound 3-Formyl Chromone

3-formyl chromone was prepared by formylation of 2-hydroxyacetophenone using the Vilsmeier–Haack reaction as reported with a slight modification (scheme 1) [18]. 2-hydroxyacetophenone (8.7 g, 0.05 mole) was added into a 500 mL clean and dry round-bottom flask, and then added 150 mL of dimethylformamide (DMF) was added. The reaction mixture was cooled to 0–5°C and phosphorus oxychloride (7.0 mL, 0.075 mole) was added dropwise at 0–10°C with continuous stirring of the reaction mixture for 30 minutes under cooling conditions. The reaction mixture was further stirred at 20–30°C for 10 hours. Finally, the thick reaction mixture was poured onto the 300-gram crushed ice and stirred for 2 hours. The light-yellow solid that precipitated out was filtered, washed with water, which was then dried to get 3-formyl chromone, and recrystallized using acetone. The yield was 82.3% and the melting point was 151°C (standard 151–153°C).

### 2.3. Synthesis of 3-(-(2-hydroxyphenylimino) Methyl)-4H-Chromen-4-One

The Schiff base ligand (SL) named 3-(-(2-hydroxyphenylimino) methyl)-4H-chromen-4-one was synthesized following the reported procedure by condensation reaction between 3-formyl chromone and 2-aminophenol in 1 : 1 stoichiometric ratio using absolute ethanol as solvent and refluxing for nearly six hours with continuous stirring at 50–60°C using a hot stage magnetic stirrer (Scheme 1) [16, 19, 20]. The progress of the reaction was monitored by TLC. Finally, the reaction mixture was concentrated to nearly 20 mL and then poured into crushed ice and stirred to get a light-yellow solid mass, which was filtered and washed with water. The obtained material was purified by recrystallization using absolute ethanol and dried in a desiccator over anhydrous calcium chloride. The yield obtained was 68.2%, and the melting point was 202°C (reported 198–200°C) [21].

### 2.4. Preparation of  Stock Solutions of Metal Ions and the Ligand Chemosensor

The solutions of metal ions were prepared by dissolving metal chloride salts of the following ions: Cu^2+^, Cr^3+^, Fe^2+^ Ni^2+^, Ca^2+^, Co^2+^, Mg^2+^, Zn^2+^, Fe^3+^, NH_4_VO_3_ (V^5+^), Mn^2+^, Hg^2+^, Pb^2+^, Ba, and Al^3+^ with 1 × 10^−2^ M concentration in double-distilled water, and the pH was between 6.20–6.50. The stock solution of the targeted ligand chemosensor was prepared with a concentration of 1.0 × 10^−2^ M by dissolving in absolute ethanol, and the pH was 7.84. The UV–vis spectra measurements were performed using a solution of 1 × 10^−3^ M concentration for the ligand compound in absolute ethanol in the range of 200–800 nm.

### 2.5. Cationic Selectivity Recognition

In this experiment, 1 mL of solution with a concentration of 1 × 10^−2^ M of each metal ion was mixed with 2 mL of the examined Schiff base ligand (SL) with a concentration of 1 × 10^−2^ M in a test tube, and the final volume of the mixture was completed to 10 mL by adding absolute ethanol. The final pH was measured at 7.37, and the room temperature was 26°C. The mixtures were left for 20 minutes at room temperature to allow complete complexation. Color changes were monitored using UV–visible spectroscopy.

### 2.6. Limit of Detection

The limit of detection (LOD) was estimated based on a UV–visible titration experiment. For this purpose, a plot of the measured absorbance intensity at a wavelength of 470 nm (in the case of Cu^2+^ and Fe^3+^) and 466 nm (in the case of V^5+^) versus the concentration of each metal ions (Cu^2+^, Fe^3+^, and V^5+^) was constructed. To perform the titration experiment for each of Cu^2+^, Fe^3+^, and V^5+^, a solution (fixed volume = 1 mL) of the ligand SL with a concentration of 0.001 M in ethanol was taken into a test tube, and to it, a series of 10 solutions (ranging from 0.01 to 1.0 mL of 0.001 M) of each metal ion in deionized water was added. After well mixing, the UV–visible spectra were scanned at room temperature in the range of 200–800 nm for each sample. The limit of detection was estimated based on the literature reported procedure [22, 23].

### 2.7. Binding Constant

The binding constant for the SL-M^n+^ complex was determined by colorimetric titration of metal with different concentrations of LS. The binding has also been verified using the aid of the Benesi–Hildebrand equation [24].

(1) $$\begin{array}{c} \frac{{\left[D\right]}_{o}}{Abs}=\left(\frac{1}{\left[A_{o}\right]}\right)\left(\frac{1}{\left[\varepsilon ka\right]}\right)+\frac{1}{\varepsilon }\text{,} \end{array}$$

where [D]_o_ is the concentration of LS, Abs refers to the absorbance of the complex at wavelength, [A]_o_ refers to the concentration of M, ka and *ε* are the molar absorptivity of the complex at *λ* and the binding constant for LS-Mn^+^. The binding constant Ka was evaluated graphically by plotting 1/[A]_o_ vs. [D]_o_/Abs.

### 2.8. Estimation of Coordination Stoichiometry

In order to estimate the binding stoichiometry between the ligand chemosensor and the metal ions, we used Job's plot method [1]. For this purpose, 0.001 mmol (0.0265 mg) of SL ligand was dissolved in 100 mL of absolute ethanol. A series of ligand solutions ranging from 0.1 mL to 0.9 mL were transferred to different flasks. Then, 0.001 mmol of each metal ion of Cu^2+^, Fe^3+^, and V^5+^ was dissolved in 10 mL of distilled water, and a series of each metal solution ranging from 0.1 mL to 0.9 mL was added to each flask of the SL ligand solution, respectively. Each mixture was completed to a volume of 10 mL by adding absolute ethanol solvent, shaken properly, and UV–visible spectra were recorded. Job's plot was then constructed by plotting the molar fraction of the chemosensor (SL) against absorbance intensity at the strongest wavelength for each metal ion, which was 456, 461, and 465 nm for Cu^2+^, Fe^3+^, and V^5+^, respectively.

### 2.9. Application of SL Chemosensor in Real Water Samples

To examine the possible utility of the tested SL chemosensor in real samples, three water samples, including distilled water, tap water from domestic water supplies, and a water sample from the Al-Aqiqwater-reservoir dam located at Al-Baha region, KSA, were collected, and each sample was spiked with a known concentration (70 *µ*M) of each Cu^2+^, Fe^3+^, and V^5+^ solution and analyzed with the SL chemosensor (140 *µ*M). All the real water samples were filtered to remove solid impurities using Whatman medium flow filter paper (Grade 1: 11 *µ*m) and centrifuged using a benchtop centrifuge at 16,000 rpm (the pH was in the range of 6.4–7.8). UV–visible absorption spectra were recorded at 470 nm in the case of Cu^2+^ and Fe^3+^ and at 466 nm in the case of V^5+^ for each sample, and the amount of Cu^2+^, Fe^3+^, and V^5+^ in each sample was calculated using the calibration curve. Each experiment was repeated three times, and the mean values were recorded.

### 2.10. Interference of Other Competing Metal Ions

To determine the possible interference from other metal ions and the selective binding affinity of chemosensor SL towards Cu^2+^, Fe^3+^, and V^5+^, UV–vis spectra were taken in the presence of other analytes. Chemosensor SL (0.0265 mg, 1 × 10^−3^ M) was dissolved in the ethanol solvent (1 × 10^−3^ M), and 70 *μ*L of each metal solution (1 × 10^−3^ M,, Cr^3+^, Ni^2+^, Ca^2+^, Co^2+^, Mg^2+^, Zn^2+^, K^+^, Mn^2+^, Hg^2+^, Pb^2+^, Ba, and Al^3+^) was taken and diluted to 10 mL with ethanol. After mixing them for a few seconds, UV–vis spectra were obtained at room temperature (25°C ± 2).

### 2.11. Preparation of Metal Complexes

#### 2.11.1. Synthesis of Dioxovanadium (V) Complexes

For preparing dioxovanadium complexes, we have used both ammonium monovanadate (NH4VO3) and potassium metavanadate (KVO3) salts. An aqueous solution (30 mL) of NH4VO3 (0.117 g, 1 mmol) or KVO3 (0.138 g, 1 mmol) was added to a hot magnetically stirred ethanolic solution (50 mL) of the synthesized Schiff base ligand 3-(-(2-hydroxyphenylimino) methyl)-4H-chromen-4-one (0.265 g, 1 mmol), and the whole mixture was stirred and heated for five hours using a hot stage magnetic stirrer. The orange precipitates thus formed were filtered, washed with methanol and dioxane, and then with diethyl ether and dried in a vacuum. The yields obtained were 76.6% and 70.3% in the cases of using ammonium monovanadate and potassium metavanadate, respectively.

#### 2.11.2. Synthesis of Fe (III) Complex

A warm ethanolic solution of ferric chloride (FeCl_3_) (1 mmol, 0.16 g) was added slowly to a hot ethanolic solution of 3-(-(2-hydroxyphenylimino) methyl)-4H-chromen-4-one (L) (2 mmol, 0.53 g) in a round-bottomed flask with shaking. The mixture was then refluxed for seven hours with continuous stirring using a magnetic hot-stage stirrer. The obtained dark red precipitate was filtered, washed many times with cold ethanol and diethyl ether, and finally dried in a desiccator over calcium chloride. The yield obtained was 81.4%.

#### 2.11.3. Synthesis of Cu (II) Complex

For the synthesis of the Cu (II) complex, we followed the reported procedure [18]. The SL ligands (0.27 g, 0.001 mol) and Cu (II) acetate (Cu (CH_3_COOH)_2_·H_2_O, 0.1 g, 0.0005 mol) were dissolved separately in 20 mL ethanol and then mixed in a round-bottomed flask. The mixture was refluxed and stirred for 6 hours using a hot-stage magnetic stirrer. The obtained brown solid precipitate was filtered, washed several times with hot ethanol and diethyl ether, and finaly dried in vacuum. The yield obtained was 77.5%.

## 3. Results and Discussion

### 3.1. Molecule Design and Preparation of the Schiff Base Ligand (SL) Chemosensor

The selected Schiff base ligand 3-(-(2-hydroxyphenylimino) methyl)-4H-chromen-4-one was successfully prepared by a one-step condensing reaction between 3-formyl chromone and 2-aminophenol, and its structure was characterized and confirmed using mass spectroscopy, IR, and ^1^H NMR spectroscopy and was similar to those reported in the literature [20, 25]. This compound is characterized by the presence of three adjacent coordination sites (the N atom of the azomethine group, the O atom of the phenolic group, and the O atom of the ketonic group), which can bind to metal ions in 1 : 1 or 1 : 2 (M: L) stoichiometric ratio easily and form different colored metal complexes with different metal ions that can be recognized easily with the naked eye [15, 25]. Moreover, this compound is soluble in ethanol, DMF, and DMSO, which are miscible with water. For this reason, we have selected this ligand (SL) to be investigated as a sensor for the presence of metal ions in water solutions.

### 3.2. Colorimetric Sensing Properties of (SL) Compound and Selectivity

**Figure 1:**
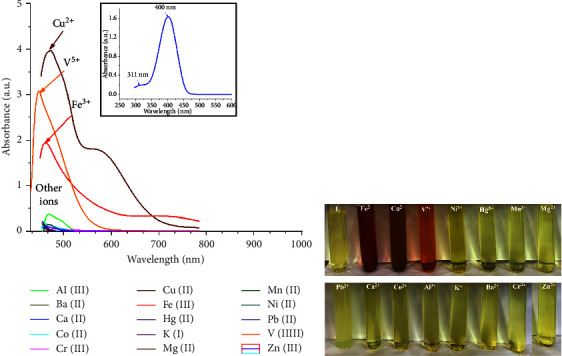
(a) UV–visible spectra of SL (1.0 × 10^−3^ M) in the presence of 1 equiv. of different metal ions (1.0 × 10^−3^ M) Cu^2+^, Cr^3+^, Fe^2+^Ni^2+^, Ca^2+^, Co^2+^, Mg^2+^, Zn^2+^, Fe^3+^, NH_4_VO_3_ (V^5+^), Mn^2+^, Hg^2+^, Pb^2+^, Ba^2+^, and Al^3+^, UV-vis. Absorption spectra of the free ligands SL (inset), (b) “naked eye” monitoring of color changes of SL (1.0 × 10^−3^ M) on adding 1 equiv. of different metal ions (1.0 × 10^−3^ M) Cu^2+^, Cr^3+^, Fe^2+^Ni^2+^, Ca^2+^, Co^2+^, Mg^2+^, Zn^2+^, Fe^3+^, NH_4_VO_3_ (V^5+^), Mn^2+^, Hg^2+^, Pb^2+^, Ba^2+^, and Al^3+^ in ethanol-H_2_O solvent mixture (9 : 1, v/v).

The UV–visible maximum absorption spectra (*λ*_max_) for the compound (SL) were recorded in absolute ethanol at room temperature with a 1 × 10^−3^ M concentration over the wavelength range of 200–800 nm. The strongest absorption band (Figure 1(a) inset) appeared in the visible wavelength period at 400 nm attributed to *n* ⟶ *π*^*∗*^ allowed transitions of the azomethine (-CH=N-) group, which is considered *λ*_max_ and used for further investigation. Another weak band at lower energy appeared at 311 nm and can be assigned to *π* ⟶ *π*^*∗*^ transitions due to *π*-orbital localization on the aromatic ring [17, 26].

To investigate the recognition ability and examine the selectivity of the prepared SL compound, several cationic metal ions (Cu^2+^, Cr^3+^, Fe^2+^, Ni^2+^, Ca^2+^, Co^2+^, Mg^2+^, Zn^2+^, Fe^3+^, NH_4_VO_3_ (V^5+^), Mn^2+^, Hg^2+^, Pb^2+^, Ba^2+^, and Al^3+^) were used. In this experiment, 1 equiv. of each metal ion was used to prepare the solutions in double-distilled water and was added to the ethanolic solution of the SL compound, and color change was monitored by the naked eye and through the recording of UV–visible absorption spectra in the range of 200–800 nm. No significant color change was noticed on the addition of the screened metal ion solutions to the tested SL chemosensor solution except for Cu^2+^, Fe^3+^, and V^5+^, which showed a significant color change from yellow to brown, red, and orange, respectively (Figure 1(b)). Moreover, it was noticed that with the addition of Cu^2+^, Fe^3+^, and V^5+^ ion solutions, there was a decrease in absorbance intensity (hypochromic effect) and an emergence of a new band in the range of 450–700 nm with the absorption maxima at 472 nm (Figure 1(a)). While on the addition of other metal ion solutions, we did not observe any effect on the absorption intensity at 472 nm (Figure 1(a)).

**Figure 2:**
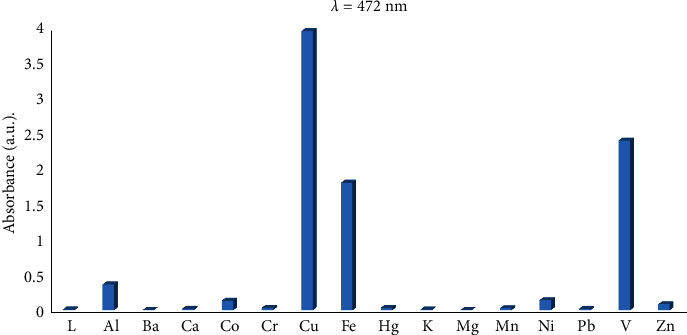
UV–visible absorption maxima.

The obtained color change, hypochromic shift, and bathochromic shift (red-shift of about 72 nm) of the UV–visible band of the free ligand may be due to deprotonation of the phenolic group in the chemosensor SL ligand and complexation of the ligand with Cu^2+^, Fe^3+^, and V^5+^ metal ions, forming colored metal complexes. Additionally, the ligand-to-metal charge transfer (LMCT) due to complexation is another factor that affects the color change [27]. The selectivity of the examined SL chemosensor towards various tested common metal ions based on absorbance intensity was plotted as a bar graph in Figure 2.

### 3.3. Estimation of Coordination Stoichiometry and Binding Modes

#### 3.3.1. Job's Plot and Binding Stoichiometry

To estimate the binding stoichiometry of the SL ligand with Cu^2+^, Fe^3+^, and V^5+^ ions, the Job plot method of continuous variation was used [11]. The molar ratio was given by

(2) $$\begin{array}{c} \frac{\left[metal\;ion\right]}{\left(\left[metal\;ion\right]+\left[SL\right]\right)}\text{.} \end{array}$$

**Figure 3:**
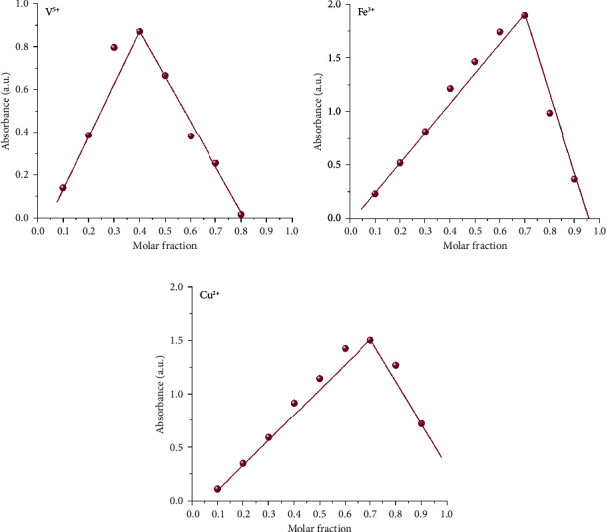
Job's plot for SL at wavelengths 456, 461, and 465 nm with Cu^2+^, Fe^3+^, and V^5+^, respectively, by UV–vis. spectra.

**Figure 4:**
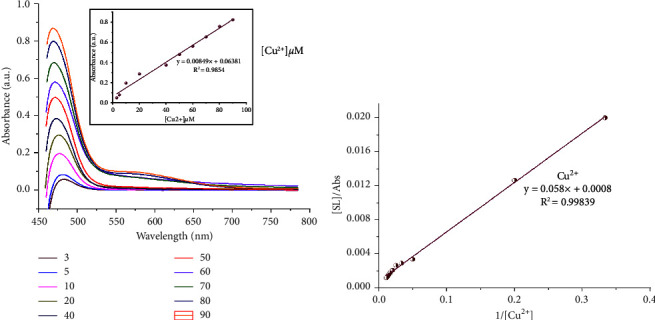
(a) UV–visible absorption spectra of SL upon the addition of different equiv. of Cu^2+^ ions with absorbance intensity of SL as a function of Cu^2+^ concentration at 470 nm (inset), and (b) Benesi–Hildebrand plot of [SL]/Abs against 1/[Cu^2+^].

**Figure 5:**
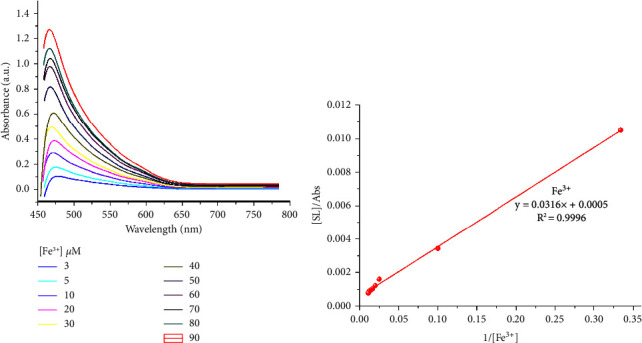
(a) UV–visible absorption spectra of SL upon the addition of different equiv. of Fe^3+^ ions with absorbance intensity of SL as a function of Fe^3+^ concentration at 470 nm (inset), and (b) Benesi–Hildebrand plot of [SL]/Abs against 1/[Fe^3+^].

**Figure 6:**
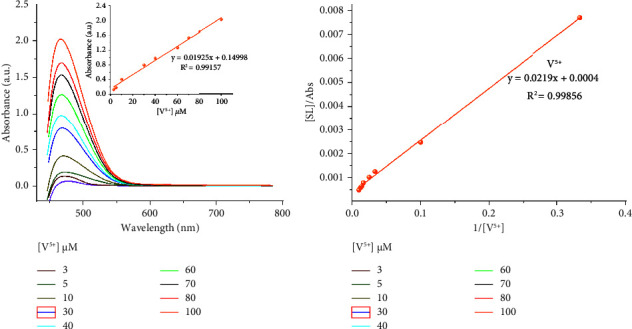
(a) UV–visible absorption spectra of SL upon the addition of different equiv. of V^5+^ ions with absorbance intensity of SL as a function of V^5+^ concentration at 470 nm (inset), and (b) Benesi–Hildebrand plot of [SL]/Abs against 1/[V^5+^].

**Figure 7:**
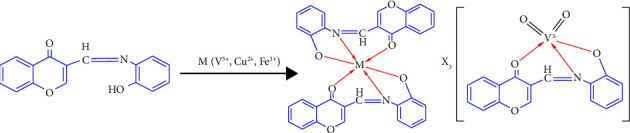
Proposed structures for the SL-Cu^2+^, SL-Fe^3+^, and SL-V^5+^ complexes.

From Job's plot (Figures 3(a)–3(c), 4(a), 4(b)–6(a), 6(b)), it is noticed that the maximum absorbance value obtained when the molar ratio was 0.66, 0.66, and 0.5 for Cu^2+^, Fe^3+^, and V^5+^, respectively, which suggests that the binding stoichiometry between the SL chemosensor and the metal ions Cu^2+^, Fe^3+^, and V^5+^ is 1 : 2 (M: L) in case of SL-Cu^2+^ and SL-Fe^3+^ complexes, while the molar ratio in the SL-V^+5^ complex was 1 : 1 in the binding stoichiometry. Accordingly, we proposed the possible complexation structure shown in Figure 7 between the SL ligand and the targeted metal ions.

#### 3.3.2. UV–Visible Titration Studies and Limit of Detection (LOD)

UV–visible titration experiments were performed in order to determine the limit of detection and further investigate the chemosensing properties of SL towards the targeted Cu^2+^, Fe^3+^, or V^5+^ metal ions. As shown in Figures 4(a)–6(a), the absorption band increased gradually for Cu^2+^, Fe^3+^, or V^5+^ ions. A calibration curve was made at 470 nm in the case of Cu^2+^, Fe^3+^, and 466 nm for V^5+^ ions. Figures 4(b)–6(b) showed a linear relationship between the absorbance intensity of SL and the concentration of Cu^2+^, Fe^3+^, or V^5+^. The values of the calculated coefficients of determination *R*^2^ = 0.9854, 0.9906, and 0.99157 in the cases of Cu^2+^, Fe^3+^, and V^5+^, respectively, which means that the adopted linear model is well adjusted to experimental results. Considering the above results, LOD (Cu^2+^) = 7.03 *µ*M, LOD (Fe^3+^) = 5.16 *µ*M, and LOD (V^5+^) = 5.94 *µ*M. The value of the binding constant (*K*_*a*_) of the SL-M^n+^ complex was evaluated with the aid of the Benesi–Hildebrand equation [17]. The plot of 1/[A]o vs [D]_o_/Abs is shown in Figures 4(b)–6(b). The binding constants of LS toward V^5+^, Cu^2+^, and Fe^3+^ were found to be 1.82 × 10^4^ M^−1^, 1.37 × 10^4^ M^−1^, and 2.01 × 10^4^ M^−1^, respectively.

### 3.4. Elucidation of SL-Cu^2+^, SL-Fe^3+^, and SL-V^5+^ Complexes Structure

In order to confirm the formation of metal complexes and the observed stoichiometric ratio for the formation of metal complexes, as well as the possible binding modes of the synthesized SL ligand with the metal ions, the metal complexes were prepared and characterized using FTIR, mass spectroscopy, UV–visible, and ^1^H NMR spectroscopy analysis.

#### 3.4.1. Mass Spectrometry Measurements

In order to prove and support the formation of metal complexes and the binding of SL with Cu^2+^, Fe^3+^, and V^5+^, we have performed a mass spectroscopy analysis. The measured (m/z) mass fractions for the free ligand and its metal complexes confirm the molecular weights according to the proposed structures. The measured mass spectrum for the free ligand showed a signal m/z 266.08 (SL + H^+^), which is equivalent to the theoretically calculated formula weight (265.16). whereas the observed mass fractions m/z at 627.5 637.92, 464.17, and 400.08, which are in line with the calculated molecular weights of 628.06, 638.356, 464.46, and 401.314 of the suggested [Cu(SL)_2_].2H_2_O, [Fe(SL)_2_].3H_2_O, K_3_[VO_2_SL], (NH_4_)_3_[VO_2_SL] complexes, respectively.

#### 3.4.2. FT-IR Investigation

The important infrared spectral data obtained for the free ligands and their Fe^3+^ and V^5+^ complexes are listed in Table 1. The characteristic strong, sharp band observed at 1602 cm^−1^ in the FT-IR spectrum of the free organic ligand (SL) is due to *ν*(C=N) vibration [15, 25]. This band suffered a hypsochromic shift (blue shift) of about 19–17 nm on complexation with metal ions and appeared in the range 1583–1585 cm^−1^. This is considered a sign of the involvement of the nitrogen atom of the azomethine group in coordination with the metal ion. Also, the band observed at 1693 cm^−1^ due to the ketonic *ν*(C=O) group of the chromone moiety in the free ligand (SL) suffered a blue shift on complex formation with metal ions and appeared in the range of 1635–1645 cm^−1^, which indicates involvement of the oxygen atom of this group in binding to metal ions [15, 25, 28]. The broadband due to the phenolic group (-OH) appeared at 3197 cm^−1^ in the spectrum of the free organic molecule (SL) and disappeared in the spectra of the metal complexes, indicating bonding of the oxygen atom after deprotonation of the phenolic group with metal ions [29]. The binding of oxygen atoms and nitrogen atoms of the ligand SL with metal ions was supported by the appearance of new bands in the range 487–489 cm^−1^ and 549–585 cm^−1^ may be due to *ν*(M-N) and *ν*(M-O), respectively, in the IR spectrum of the metal complexes [1]. Therefore, the synthesized SL ligand is considered a tridentate ligand and has the ability to bind through ONO atoms with metal ions. In the IR spectrum of vanadium complexes, a sharp band was observed in the range 911–960 cm^−1^ and another broadband in the region 845–830 cm^−1^ can be assigned to *ν*_asym_ and *ν*_sym_ vibrations of cis-VO_2_ groups [30]. Moreover, a broadband was observed in the range 3258–3400 cm^−1^ in the IR spectra of metal complexes that can be attributed to the presence of water molecules [31].

#### 3.4.3. ^1^H-NMR Spectra

In order to confirm the complexation of the SL ligand with Fe^3+^ and V^5+^, we have recorded the ^1^H-NMR spectra of the free ligand and its metal complexes. The observations showed a downfield proton appeared at 12.04 ppm and may be assigned to the phenolic –OH group, and an azomethine (HC=N) proton appeared at 8.15 ppm [32]. The peak due to the phenolic group disappeared in the spectra of Fe^3+^ and V^5+^ complexes, indicating deprotonation of the OH group and bonding to the metal ions [29]. Moreover, the peak due to azomethine proton suffered a chemical shift and appeared at 8.945, 8.68, and 8.05 in the spectra of the (NH_4_)_3_[VO_2_SL], K_3_[VO_2_SL], and [Fe(SL)_2_].3H_2_O, respectively, due to the involvement of the nitrogen atom of the azomethine group in coordination with metal ions [29].

### 3.5. The Suggested Binding Mechanism

According to the observations of Job's plot, UV–visible titration, mass spectra, FTIR measurements, and NMR spectral analysis, the binding possibility of the tested Schiff base ligand (SL) to the targeted metal ions can be suggested, as shown in the proposed feasible structures of the complexes with general formulae [Cu(SL)_2_].2H_2_O, [Fe(SL)_2_].3H_2_O (i.e., binding in 1 : 2 (M: L) molar ratio in the case of copper and iron) and K_3_[VO_2_SL] or (NH_4_)_3_[VO_2_SL] (i.e., binding in 1 : 1 (M: L) molar ratio in the case of vanadium), as shown in Figure 7 (a & b). The synthesized Schiff base ligand SL is coordinated to metal ions through the nitrogen atom of the azomethine group (-CH=N-) and the oxygen atom of the phenolic group (-OH) with deprotonation, and the oxygen atom of the ketonic group (C=O). These coordination sites are similar to those reported in the literature for similar ligands containing different metal ions and forming distorted octahedral geometry [15, 25, 28].

### 3.6. Interference from Other Metal Ions

**Figure 8:**
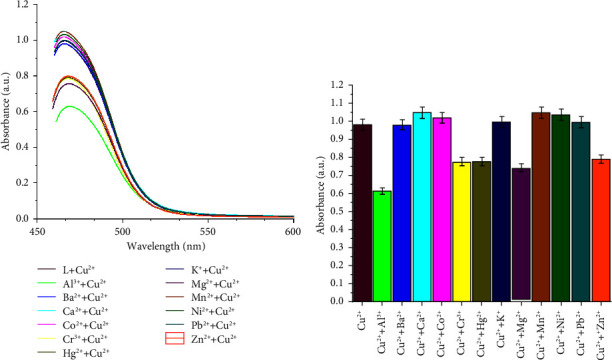
(a, b) Interference studies for the selectivity of the chemosensor SL towards Cu^2+^ (70 *μ*M) in the coexistence of other cationic species (70 *μ*M).

**Figure 9:**
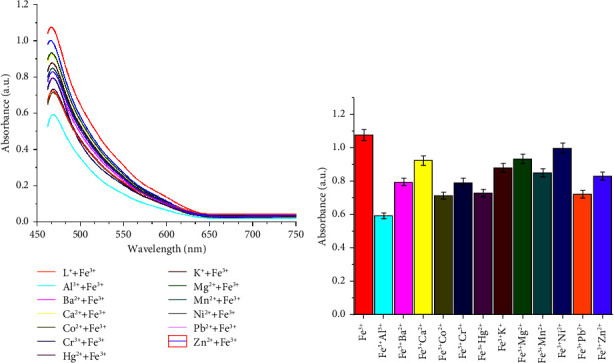
(a, b) Interference studies for the selectivity of the chemosensor SL towards Fe^3+^ (70 *μ*M) in the coexistence of other cationic species (70 *μ*M).

**Figure 10:**
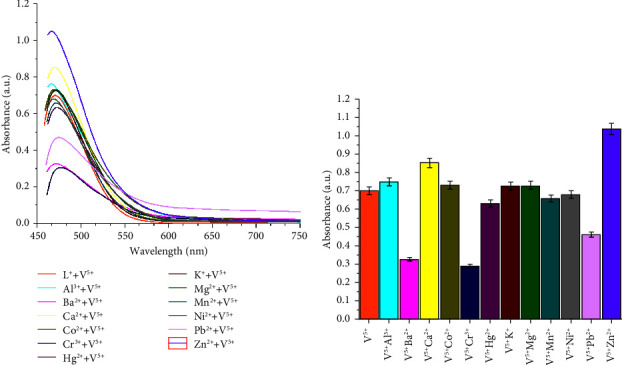
(a, b) Interference studies for the selectivity of the chemosensor SL towards V^5+^(70 *μ*M) in the coexistence of other cationic species (70 *μ*M).

The interference of the coexisting ions during the practical utility of the examined chemosensor SL for Cu^2+^, Fe^3+^, and V^5+^ ion detection in the presence of different metal ions such as Cr^3+^, Fe^2+^, Ni^2+^, Ca^2+^, Co^2+^, Mg^2+^, Zn^2+^, Mn^2+^, Hg^2+^, Pb^2+^, Ba, and Al^3+^ was investigated. For this purpose, a competitive titration experiment was performed, and each of the interfering metal ions was added separately to the solution of SL- Cu^2+^, SL-Fe^3+^, or SL-V^5+^, and the resulting solutions were scanned for UV–vis absorption spectra, and the results are expressed as absorbance intensity versus the composition of the sensing solution (Figures 8–10). It is obvious that, irrespective of initial intensity, there was no significant effect observed in the presence of other metal ions. These results clearly show the high binding affinity of the synthesized chemosensor SL towards Cu^2+^, Fe^3+^, and V^5+^ compared to other competitively used metal ions and had noticeable selectivity towards these metal ions. Moreover, to examine the stability of the targeted prepared SL chemosensor, the solutions of the SL-Cu^2+^, SL-Fe^3+^, and SL-V^5+^ complexes were kept for a period of one day (24 h), and the UV–visible spectra were recorded every 8 hours. The observations showed that the intensity of UV absorbance remained constant over the entire period, which is a sign of the stability of the examined SL chemosensor.

### 3.7. Application of a SL Chemosensor for Detection of Metal Ions in Real Water Samples

To examine the practical application of the synthesized Schiff base chemosensor for the detection of Cu^2+^, Fe^3+^,or V^5+^ metal ions, experimental tests were performed with distilled and real water samples taken from tap water of domestic water supply and from the Al-Aqiq water reservoir dam located at Al-Baha region, Saudi Arabia. Each water sample was spiked with a known amount of Cu^2+^, Fe^3+^, or V^5+^ (70 µM), and then their concentrations were estimated from the calibration curve. The observations (Table 2) showed good values for the recovery of the metal ions, indicating the ability of the examined SL chemosensor to successfully detect Cu^2+^, Fe^3+^, or V^5+^ metal ions in real water samples.

The difference between the known concentration spiked into the sample and the metal ion detected using the examined sensor may be due to the formation of stable metal complexes with Cu^2+^, Fe^3+^, or V^5+^ metal ions.

The real water samples analysis data (Table 2). The calculated recoveries for known amounts of Cu^2+^, Fe^3+^, or V^5+^ added ranged from 400.1–657.0%, 42.85–100%, and 57.14–97.14%, respectively. These results indicated that SL could be suitable and useful for the sensitive detection of Cu^2+^, Fe^3+^, or V^5+^ in real water samples with good precision and accuracy in the case of Cu^2+^ and V^5+^ and moderate precision for Fe^3+^ ions.

### 3.8. Comparison with Other Studies

There are many reported studies that have identified chromone-based Schiff base molecules that are used as colorimetric probes towards some metal ions. Fan et al. reported the use of a novel and simple Schiff base receptor based on a chromone derivative called 7-methoxychromone-3-carbaldehyde-(indole-3-formyl) hydrazone as a selective and sensitive probe for Al^3+^ with colorimetric and fluorescent responses [33]. The reported molecule had a sensing limit for recognizing aluminum ions as low as 1.0 × 10^−7^ M, which is sufficient for sensing Al^3+^ that may be available in environmental and biological systems.

Another chromone-based colorimetric sensor was developed and synthesized by the condensation reaction between 3-formyl-6-methylchromone and 2-aminobenzamide for highly selective detection of Cu^2+^ ions in semiaqueous media, which showed sensing ability for copper ions with different concentrations ranging from 10^−3^ to 10^−7^ in aqueous solutions and showed excellent applicability in real water samples [34]. Colorimetric chemosensors based on the Schiff base 2-hydroxy-5-[(2-hydroxy-1-naphthyl) methylideneamino]benzoic acid have been reported to exhibit high selectivity and sensitivity for detecting Cr^3+^, Cu^2+^, Fe^3+^, and Al^3+^ ions simultaneously in DMF/H_2_O (v/v = 1/1) solution with LOD of 3.37 × 10^−7^ M, 4.65 × 10^−7^ M, 3.58 × 10^−7^ M, and 4.89 × 10^−7^ M, respectively [32]. A chromone-based azomethine chemosensor synthesized by the condensation reaction of 3-formylchromone with 2,6-pyridinedicarbohydrazide was successfully used as an immediate and naked-eye sensor for Cu^2+^, Zn^2+^, and CN^−^ ions in aqueous media with excellent accuracy and precision with detection limits of 5.50 × 10^−7^, 8.70 × 10^−7^ and 1.56 × 10^−6^ M, respectively, which are lower than the permissible levels recommended by WHO for safe drinking water [24]. A chromone-based Schiff base molecule synthesized by the condensation reaction between 3-formyl chromone and octopamine was reported by Lee and Kim as sensor probe for Cu^2+^ ions only by fluorescence quenching with a LOD of 3.95 × 10^−6^ M [35]. Compared with the abovereported chromone-based Schiff base colorimetric sensors, our synthesized chromone-based Schiff base sensor can be considered selective for detecting three metal ions, namely Cu^2+^, Fe^3+^, and V^5+^ ions, in aqueous media with excellent accuracy and precision with detection limits of 3.322 × 10^−5^ M, 2.065 × 10^−5^ M, and 1.782 × 10^−5^ M, respectively, which are lower than the permissible level recommended by WHO for safe drinking water.

## 4. Conclusion

We have synthesized the Schiff base ligand 3-(-(2-hydroxyphenylimino) methyl)-4H-chromen-4-one (SL) and examined it as a chemosensor for the detection of metal ions in aqueous solutions. The tested compound (SL) exhibited a remarkable selectivity and sensitivity response toward three metal cations: Cu^2+^, Fe^3+^, and V^5+^. Experimental results showed 2 : 1 (SL:M) binding stoichiometry in the case of SL-Cu^2+^ and SL- Fe^3+^ complexes, while 1 : 1 stoichiometry was observed in the SL-V^5+^ complex. The LOD and bending constant were found to be (7.03 *µ*M, 1.37 × 10^4^ M^−1^), (5.16 *µ*M, 2.01 × 10^4^ M^−1^), and (5.94 *µ*M, 1.82 × 10^4^ M^−1^) for Cu^2+^, Fe^3+^, and V^5+^, respectively.

## Figures and Tables

**Table 1:** Important IR spectroscopic data (cm^−1^) of the ligands (SL) and their Cu^2+^, Fe^3+^, and V^5+^ complexes.

Compounds	Ʋ (H_2_O)	Ʋ (OH)	Ʋ (C=O)	Ʋ (C=N)	V=O	Ʋ (M-N)	Ʋ (M-O)
SL	—	3197	1639	1562	—	—	—
[Fe(SL)_2_]. 3H_2_O	3258	—	1658	1585	—	487	549
[Cu(SL)_2_]. 2H_2_O	3197	—	1655	1583	—	487	562
(MH_4_)_3_ [VO_2_SL]	3362	—	1645	1584	911	488	581
					963		
K_3_ [VO_2_SL]	3400	—	1656	1583	960	489	585

**Table 2:** Analytical results for real water sample applications.

Metal ion	Sample	Metal ion added (*μ*M)	Metal ion found (*μ*M)	Recovery %	RSD%
Cu^2+^	Tap water	70	280	400.1% ± 0.44	0.11
	Al-Aqiq dam water	70	460	657.0% ± 0.86	0.13
	Distilled water	70	400	571.0% ± 0.45	0.08

Fe^3+^	Tap water	70	40	57.14% ± 0.40	0.70
	Al-Aqiq dam water	70	30	42.85% ± 0.65	1.52
	Distilled water	70	70	100% ± 0.00	0.00

V^5+^	Tap water	70	40	57.14% ± 0.75	1.32
	Al-Aqiq dam water	70	65	92.85% ± 0.36	0.39
	Distilled water	70	68	97.14% ± 0.90	0.93

## Data Availability

All the data generated or analyzed during this study are included in the article.
